# Unusual pectoral apparatus in a predatory dinosaur resolves avian wishbone homology

**DOI:** 10.1038/s41598-021-94285-3

**Published:** 2021-07-19

**Authors:** Andrea Cau, Vincent Beyrand, Rinchen Barsbold, Khishigjav Tsogtbaatar, Pascal Godefroit

**Affiliations:** 1 Unaffiliated, 43125 Parma, Italy; 2 European Synchrotron Radiation Facility, 38043 Grenoble, France; 3 Department of Anthropology, Universitat Zurich, Winterthurerstrasse 190, 8057 Zurich, Switzerland; 4 Paleontological Center, Mongolian Academy of Sciences, Ulaanbaatar, 210-351 Mongolia; 5 Institute of Paleontology, Mongolian Academy of Sciences, 15160 Ulaanbaatar, Mongolia; 6 Directorate Earth & History of Life, Royal Belgian Institute of Natural Sciences, 1000 Brussels, Belgium

**Keywords:** Palaeontology, Palaeontology

## Abstract

The furcula is a distinctive element of the pectoral skeleton in birds, which strengthens the shoulder region to withstand the rigor of flight. Although its origin among theropod dinosaurs is now well-supported, the homology of the furcula relative to the elements of the tetrapod pectoral girdle (i.e., interclavicle *vs* clavicles) remains controversial. Here, we report the identification of the furcula in the birdlike theropod *Halszkaraptor escuilliei*. The bone is unique among furculae in non-avian dinosaurs in bearing a visceral articular facet in the hypocleideal end firmly joined to and overlapped by the sternal plates, a topographical pattern that supports the primary homology of the furcula with the interclavicle. The transformation of the interclavicle into the furcula in early theropods is correlated to the loss of the clavicles, and reinforced the interconnection between the contralateral scapulocoracoids, while relaxing the bridge between the scapulocoracoids with the sternum. The function of the forelimbs in theropod ancestors shifted from being a component of the locomotory quadrupedal module to an independent module specialized to grasping. The later evolution of novel locomotory modules among maniraptoran theropods, involving the forelimbs, drove the re-acquisition of a tighter connection between the scapulocoracoids and the interclavicle with the sternal complex.

## Introduction

**Figure 1:**
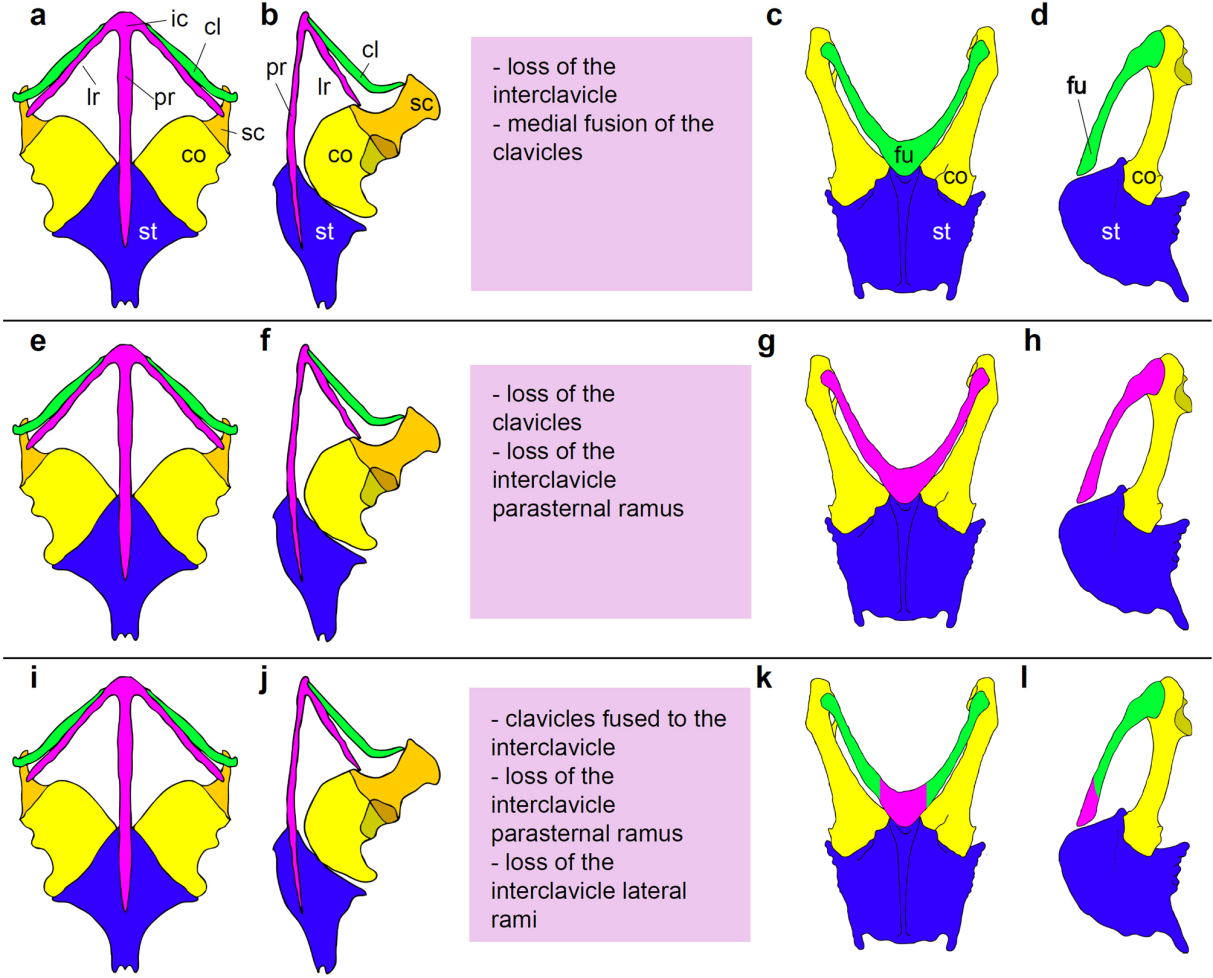
The alternative hypotheses on the homology of the avian furcula. Comparison between the pectoral apparatus of non-avian reptiles (left) and birds (right). The furcula has been alternatively considered homologous to the clavicles (**a–d**), the interclavicle (**e–h**), or as a compound bone including elements from both the clavicles and the interclavicle (**i–l**). Generalized pectoral apparatus in a non-avian reptile in ventral view (**a**,**e**,**i**) and in left anterolateral view (**b**,**f**,**j**); in the middle, list of the evolutionary changes inferred along the avian stem lineage following the selected hypothesis; generalized pectoral apparatus in a bird (scapula omitted for clarity) in ventral view (**c**,**g**,**k**) and in left lateral view (**d**,**h**,**l**). *cl* clavicle, *co* coracoid, *fu* furcula, *ic* interclavicle, *lr* lateral ramus of interclavicle, *pr* parasternal ramus of interclavicle, *sc* scapula, *st* sternum. Drawing by AC.

The origin of the peculiar biology of birds has been investigated at several levels and from different functional and phylogenetic perspectives^1–10^. The evolution of the active flight of birds has been subjected to hierarchically nested analyses, which have reconstructed the sequence of adaptive regimes that shaped the avian *bauplan*^1–3,8,9^, have focused on the origin of locomotor modularity^4^, and have discussed the emergence of the anatomical novelties involved in powered flight^5–7,10^. In birds, the furcula (or wishbone) is a peculiar unpaired dermal element of the pectoral apparatus that articulates to each of the scapulocoracoids, working as a strut between the shoulders^11^. Together with the scapula and the coracoid, the furcula also participates in the formation of the *canalis triosseus*, which houses a strong tendon connecting the supracoracoideus muscles to the humerus; this system is responsible for lifting the wings during the flight recovery stroke^4,6–9,12,13^. The homology of the furcula relative to the elements of the pectoral girdle in non-avian tetrapods is debated^14–30^. The ancestry of the avian furcula from the homonymous element widespread in non-avian theropods is well-supported^14–20^, indicating that this bone evolved well-before the origin of avian flight among bipedal ground-dwelling dinosaurs. The wishbone has alternatively been considered as a neomorphic condition resulting from the medial fusion of the clavicles^18,21–24^, as homologous to the reptilian interclavicle^22,23^, or as a compound element including both the clavicles and the interclavicle (forming, respectively, the two epicleideal processes contacting the scapula and the median hypocleideum)^25,26^ (Fig. 1). It is usually assumed that dinosaurs retained the clavicles all along the avian stem and lost the interclavicle during their earliest evolution^18,21–24^. Unfused clavicles are documented in both the ornithischian^29,30^ and sauropodomorph^22,24^ lineages, with at least the latter group occasionally retaining a rod-like interclavicle^22^. Regardless its homology, the origin of the furcula is thus firmly constrained close to the divergence of theropods from the other dinosaurs^18^. Yet, the developmental basis of wishbone formation relative to the pectoral complex of extant reptiles is more controversial^23^. Embryological evidence has dismissed a compound origin of the furcula and has provided equal support for the competing hypotheses on furcular homology with, alternatively, the clavicle and the interclavicle^23,25,26^. The two scenarios differ in the sequence of events leading to the theropod furcula from the plesiomorphic archosaurian pectoral complex^18,23^.

The immediate sister taxa of theropods have not provided unambiguous evidence in support of one scenario over the other. Some Triassic sauropodomorphs show that an incipient furcula-like pattern was present among early dinosaur clavicles^24^. Yet, such condition is likely an archosaurian plesiomorphy and cannot inform on the adaptive regime leading to the furcula^22–24^. The retention in sauropods of distinct clavicles coupled with the presence in these dinosaurs of the interclavicle bearing incipient epicleideal processes challenge the homology between the furcula and the clavicles and might instead support the origin of the wishbone from the interclavicle^22^.

The topographic relationships between the clavicles, the interclavicle and the scapulocoracoid elements are extremely variable in extinct and extant amniotes^25,26^: therefore, the epicleideal articulation between the furcula and the scapula is not a solid criterion for inferring wishbone primary homology^31^. On the contrary, in all main lineages of Amniota the sternum consistently articulates with the posterior (parasternal) ramus of the unpaired interclavicle but does not articulate with the medial extremities of the paired clavicles which—under the “clavicular hypothesis”—may constitute the precursors of the hypocleideal end of the furcula^26^. The presence and topography of a furcular-sternal relationships in the Mesozoic relatives of birds might therefore represent the most conservative test for discriminating between the two competing scenarios for the origin of the furcula. Unfortunately, the fossil record of the sternum and its contribution to the pectoral complex in non-avialan theropods is largely incomplete and biased by taphonomic and developmental factors^18,21,22^.

**Figure 2:**
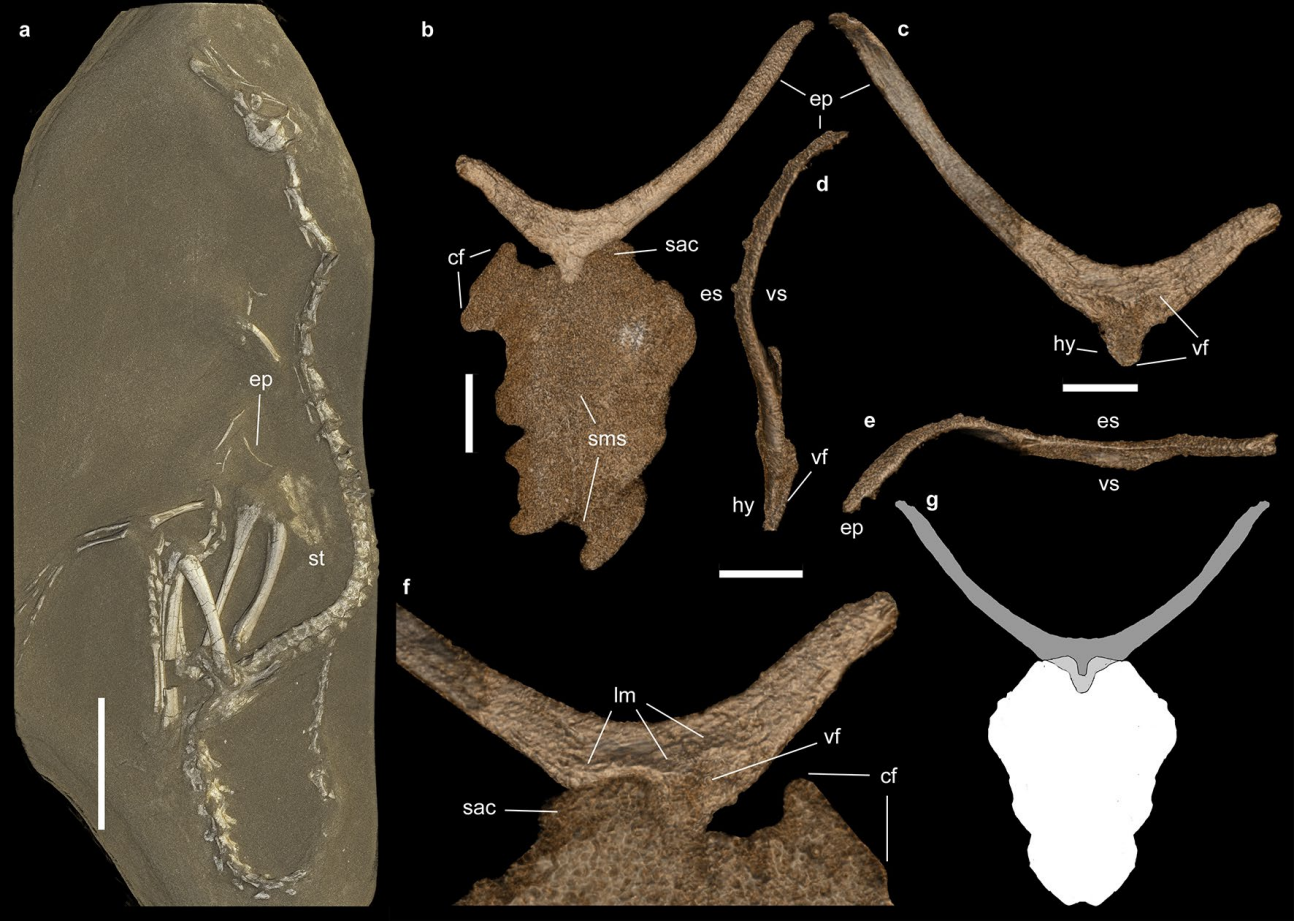
The furcula-sternum complex in *Halszkaraptor escuilliei* MPC D-102/109. (**a**) Exposed elements of *H. escuilliei* holotype. (**b**) Furcula and sternum in ventral view. (**c**) Furcula in dorsal (visceral) view. (**d**) Furcula in left lateral view. (**e**) Furcula in anterior (proximal) view. (**f**) Detail of the sternofurcular articulation in dorsal (visceral) view. (**g**) Reconstruction of the furcula-sternum complex in dorsal (visceral) view. Dark grey, exposed furcula; light grey, posterior region of furcula overlapped by the sternal plates; white, sternal plates. *cf* coracoid facet, *ep* epicleideum, *es* “external” side of the bone, *hy* hypocleideum, *lm* lipped margin of visceral fossa, *sac* sternal anterior end covering the furcula, *sms* sternal plates midline suture, *st* sternal plates, *vf* visceral fossa, *vs* “visceral” side of the bone. Scale bars: 70 mm (**a**), 8 mm (**b**), 6 mm (**c–e**). Visualization by VB using Volume Graphics vers. 2.2. (Heidelberg, Germany: https://www.volumegraphics.com).

Here, we report the identification of additional elements of the pectoral apparatus, including the furcula, in the only known specimen of the bird-like theropod *Halszkaraptor escuilliei*^32^ (Fig. 2), and emend the diagnosis of this taxon. We compare the pectoral apparatus of *Halszkaraptor* to those of other sauropsids (including birds), and discuss the origin and the primary homology of the furcula. In particular, we compare the pectoral apparatus of *H. escuilliei* with that of an extant non-avian reptile (the lepidosaurian *Varanus storri*), based on high-resolution µCT data obtained from a complete and articulated specimen housed in the RBINS (see “Methods” section). Although the crocodiles are the closest extant relatives of birds^1,4,23^, their pectoral apparatus is markedly derived relative to the ancestral diapsid and archosaurian conditions because it lacks the clavicles^22,23,25,26^, and thus is inadequate in addressing the alternative hypotheses on the primary homology of the avian furcula.

## Results

### Systematic palaeontology

Dinosauria Owen

Theropoda Marsh

Dromaeosauridae Matthew and Brown

Halszkaraptorinae Cau et al.

*Halszkaraptor escuilliei* Cau et al.

#### Emended diagnosis

(Autapomorphies marked by asterisk) Platyrostral premaxilla that forms 32% of snout length and bears 11 teeth*; extensively pitted prenarial body of premaxilla; large foramen on lateral surface of base of nasal process of premaxilla*; first two maxillary teeth smaller than either premaxillary and subsequent maxillary teeth, and slightly procumbent; external naris posterior to the premaxillary oral margin; rod-like jugal with an ascending process excluded from the orbital margin that forms only 10% of the postorbital bar*; rod-like ventral ramus of the postorbital; 22 presacral vertebrae; neck forms 50% of snout–sacrum length*; absence of epipophyses; ridge-like cervical neural spines restricted to the 2nd–5th vertebrae; postzygapophyses on cervicals 2–5 are fused medially and form single lobate processes*; pleurocoels restricted to cervicals 7–9; tuber-like neural spines in tail are restricted to the 1st–3rd vertebrae; proximal-most chevrons large and pentagonal; transition point in 7th–8th caudals; furcula with tongue-like hypocleideum; visceral surface of furcula with a bilobed facet for anteromedial processes of sternum*; 3rd finger longer than 2nd; medial surface of preacetabular processes contacting medially dorsal to the anterior sacral neural spines; distal third of metatarsal IV shaft bear a low ridge along its flexor margin; elongate pedal phalanx III-1 is 47% of the length of metatarsal III .

#### Description of the pectoral apparatus of *Halszkaraptor escuilliei*

**Figure 3:**
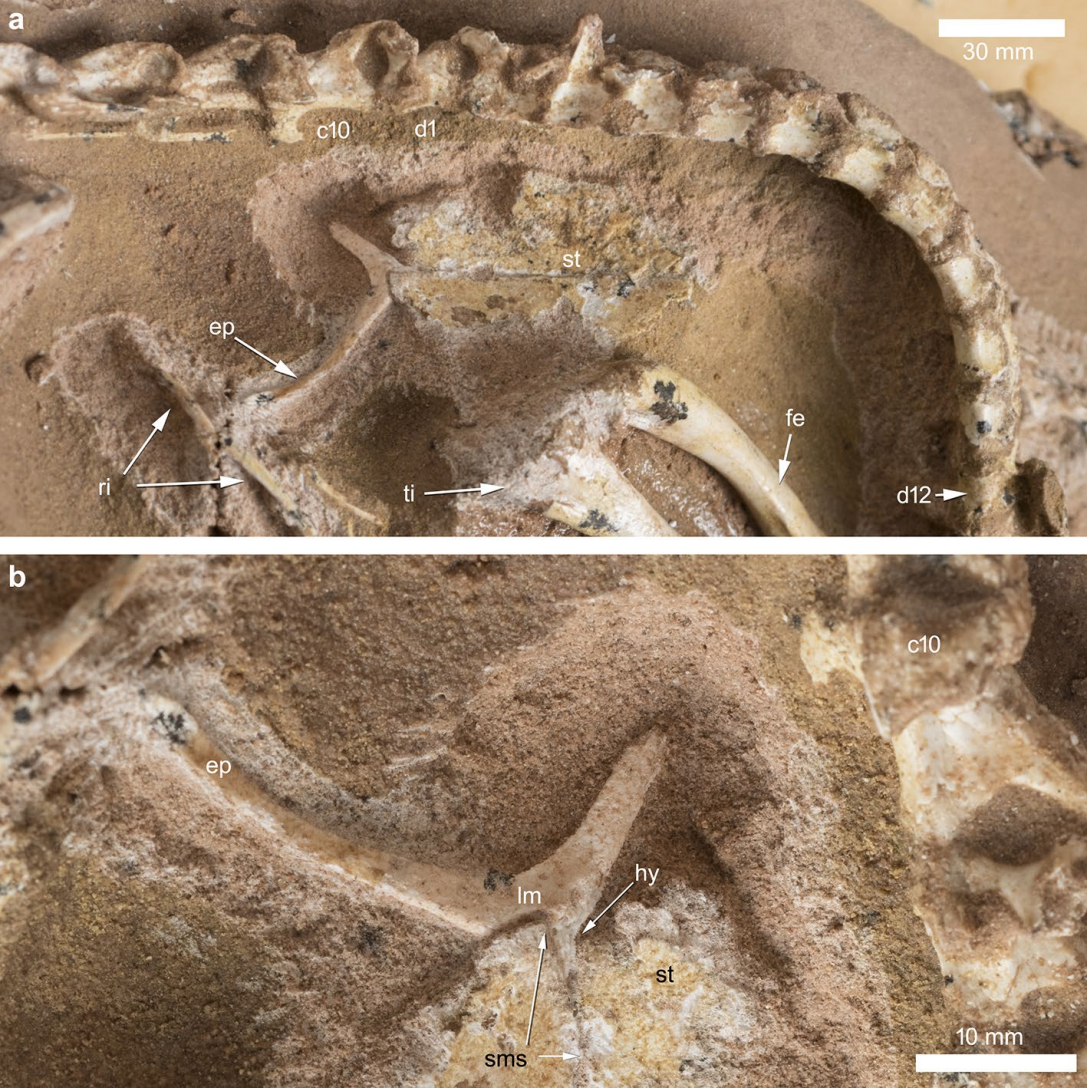
Mechanically prepared pectoral region of *Halszkaraptor escuilliei* MPC D-102/109. (**a**) Exposed sternum and furcula in visceral view. (**b**) Detail of the sternal articular surface of the furcula in left laterovisceral view. *c10* 10th cervical vertebra, *d1* 1st dorsal vertebra, *d12* 12th dorsal vertebra, *ep* epicleideum, *fe* femur, *hy* hypocleideum, *lm* lipped margin of the sternal facet, *ri* ribs, *sms* open midline suture between the sternal plates, *st* sternal plate, *ti* tibia. Scale bar: 30 mm (**a**), 10 mm (**b**). Photos by T. Hubin.

The sternum and part of the left scapula were the only elements visible externally prior to mechanical preparation^32^. The furcula, almost completely embedded in the rock matrix and not exposed except for the lateral margin of the left epicleideum (Fig. 2a), has been identified through propagation X-ray phase-contrast synchrotron microtomography and mechanically prepared (Fig. 3a). It is preserved in its original anatomical position, ventral to the presacral vertebral series near the cervico-dorsal transition, medial to both forelimbs and anterior to the sternal plates^18^. The scapulae and coracoids suffered the most significant disarticulation in an otherwise articulated skeleton. Synchrotron microtomography revealed that the right scapula is almost completely preserved inside the rock matrix. It is an elongate and slender element lacking any distinct process or expansion at its extremities. The two coracoids are represented by a couple of strut-like elements adjacent to the anterior end of each scapula. Similar to the articular ends of the long bones, we suggest that the extremities of both scapulae and coracoids were scavenged by saprophagous invertebrates immediately after the death of the animal^32^. The furcula is only missing the distal half of the right epicleideum and is adjacent to the anterior end of the conjoined sternal plates. The epicleideal rami are slender, comparable to the sternum in length, anteroposteriorly compressed and bowed posterodorsally in lateral view (Fig. 2b–e). Together, the two epicleideal rami form an angle of 95°. The epicleideal apices are scarred by a series of sulci marking the ligamental connection with the scapular acromion^16,18^. The wishbone bears a tongue-like hypocleideum projected posteriorly and overlapped by the anterior margin of the sternal complex (Fig. 2b–d). The dorsal (visceral) surface of the furcular body bears a distinct bilobate fossa, bound anteriorly by a lipped margin, which also excavates the hypocleideum (Fig. 2c,d,f). The anterior end of the left sternal plate lodges in the left half of the bilobate furcular fossa (the anterior end of the right sternal plate is not preserved), showing that the latter is an articular surface and not a pneumatic recess (Figs. 2c,f, 3b). The two sternal plates are trapezoidal in ventral view, with the longest axis directed anteroposteriorly. The two plates are unfused medially and separated by a narrow gap. The outer margin of the bones is mostly eroded away, and only the coracoidal facet of the right plate and the sternal process of the left plate are preserved. The preserved coracoidal facet is straight and forms an angle of about 45° with the anteroposterior axis of the sternum. The preserved sternal facet articulates with the corresponding left half of the bilobed fossa of the furcula.

## Discussion

**Figure 4:**
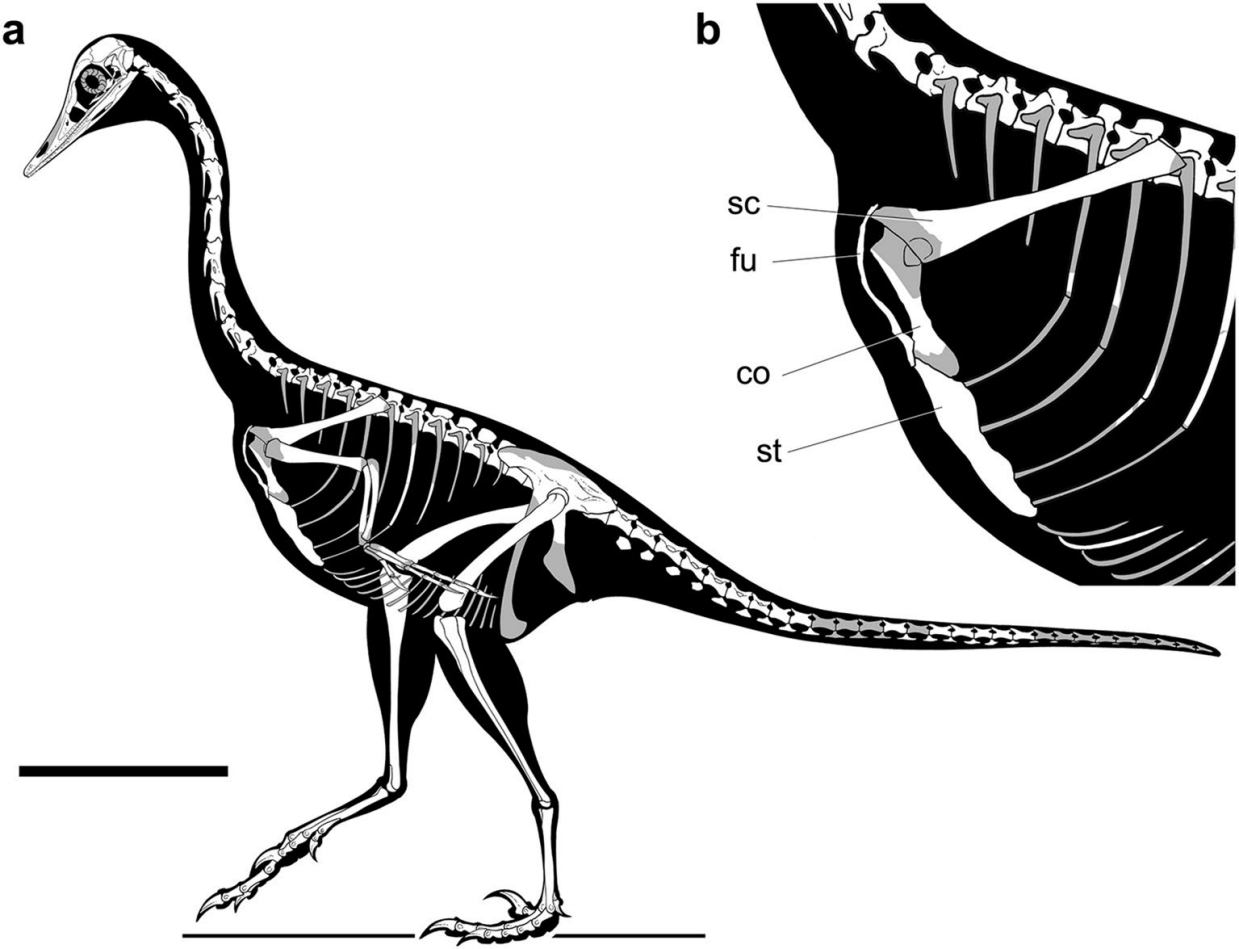
Updated reconstruction of the skeleton of *H. escuilliei*. (**a**) Whole skeleton in left lateral view; (**b**) detail of the pectoral apparatus (left forelimb removed). Missing elements indicated in grey. *co* coracoid, *fu* furcula, *sc* scapula, *st* sternum. Scale bar: 10 cm. Drawing by M. Auditore.

The presence of a distinct joint surface for the sternal plates in the furcula (still occupied by the left sternal plate) and the perfect alignment of the anteroposterior axis of the hypocleideum with the mid-line of the conjoined sternal plates despite scapulocoracoid disarticulation dismiss the possibility that post-mortem processes are responsible for the furcular-sternal contact (Fig. 2g). The information obtained combining the CT-scan data with further mechanical preparation of the specimen has provided the basis for an updated reconstruction of the skeletal anatomy of *Halszkaraptor escuilliei* (Fig. 4). In particular, several features support a peculiar morphology of the pectoral apparatus, divergent from that of most maniraptoriform theropods^18,28,33,34^ and which recalls that of pygostylian birds^34–37^ (Fig. 4b). The firm furcular-sternal articulation (Figs. 2, 3) constraints the orientation of the posteroventral surface of the furcula as paralleling the ventral surface of the sternum. The significant elongation of the epicleideal rami (Fig. 2), combined with their distinct proximodorsal curvature, provides the basis for inferring the position of the glenoid region relative to the sternal surface, and suggests a significant dorsoventral gap between the glenoid and the sternum. The latter interpretation is further supported by the partially preserved coracoids (revealed by CT-scanning), which are proximodistally elongate, and thus recall the strut-like condition acquired by flying birds^34,36–38^ more than the quadrangular or plate-like coracoids of most theropods^15–21,33^. Using the epicleideal ramus of the furcula and the coracoidal facet of the sternum as reference elements, we infer that the coracoid was more elongate proximodistally than wide transversely, and oriented perpendicular to the main axis of the scapula. We conclude that the pectoral region of *Halszkaraptor* was relatively deep, with the glenoid articulation placed approximately at mid-height between the anteroposterior axis of the thoracic vertebral column and the ventral sternal surface.

**Figure 5:**
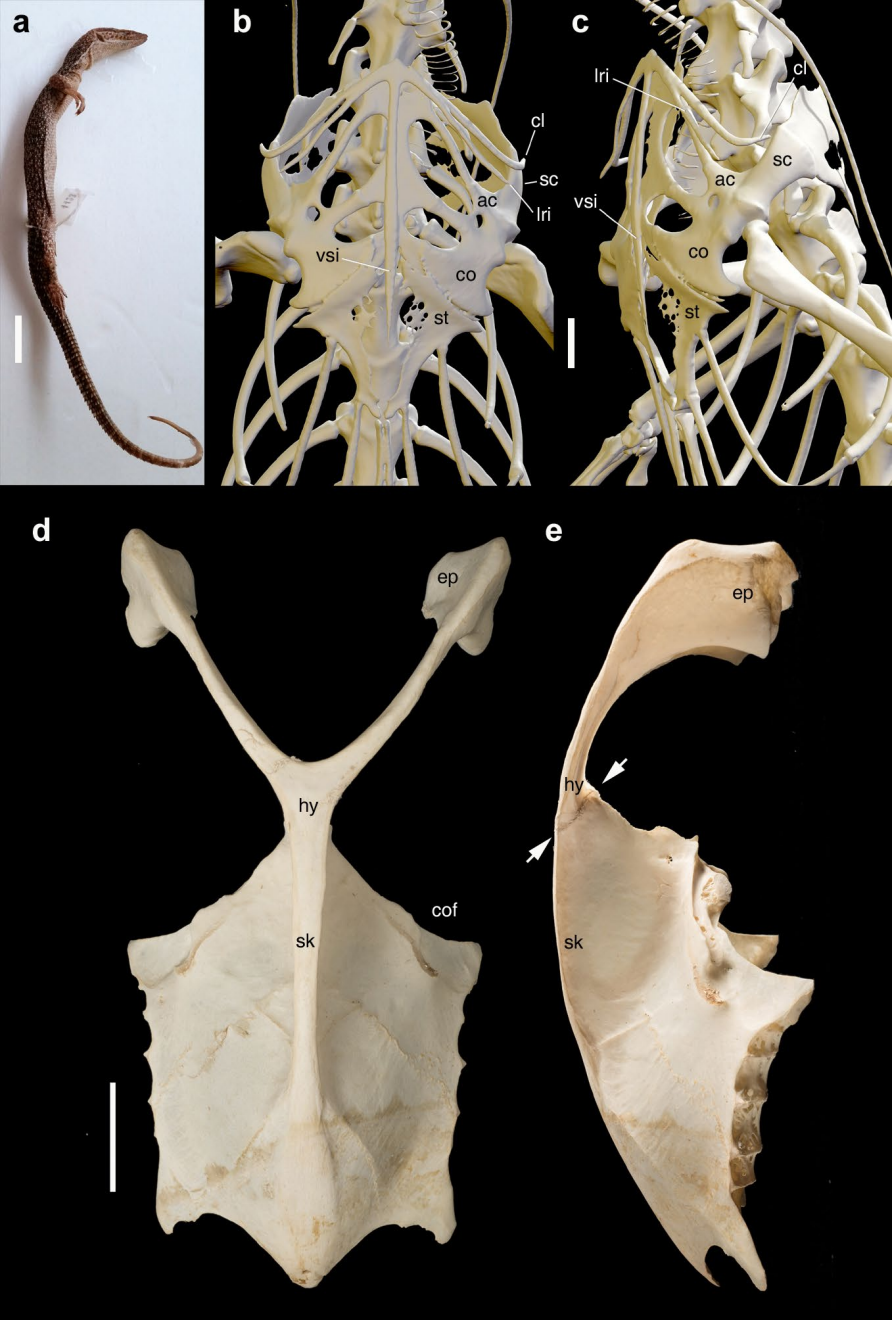
The interclavicle-sternum complex in reptiles and birds. (**a–c**) *Varanus storri* BE-RBINS-VER-10900. (**a**) The specimen subjected to µCT scanning. (**b,c**) close-up of the digitally reconstructed pectoral complex in ventral view (**b**) and oblique lateroventral view (**c**). Note the positions of clavicle and interclavicle lateral ramus relative to the acromial region, and the visceral overlap of the sternum relative to the posterior end of the interclavicle. (**d,e**) sterno-furcular complex of *Pelecanus crispus* BE-RBINS-VER-83834 in ventral view (**d**) and lateral view (**e**). Arrows in (**e**) mark the dorsoventral extent of the hypocleideum-sternal articular surface. Note the visceral overlap of the sternum relative to the hypocleideum.* ac* acromion, *cl* clavicle, *co* coracoid, *cof* coracoid facet, *ep* epicleideum, *hy* hypocleideum, *lri *lateral ramus of interclavicle, *sc* scapula,* sk* sternal keel, *st *sternum, *vsi *ventral (parasternal) ramus of interclavicle. Scale bars: 20 mm (**a**), 2 mm (**b,c**), 30 mm (**d,e**). Photos in (**a,d,e**) by T. Hubin. Visualization in (**b,c**) performed by J. Brecko using Dragonfly vers. 4.0 for Windows (Object Research Systems Inc., Montreal, Canada, 2020: http://www.theobjects.com/dragonfly).

The furcula of *Halszkaraptor* recalls both morphologically and topographically the interclavicle of the non-avian diapsid reptiles^23,26^ (Fig. 5a–c): (1) it is a single median element which is proximally bifurcated and distally spatulate; (2) it articulates posteriorly with (and is viscerally overlapped by) the ventromedial apex of the sternal plates. Combined with evidence gleaned from pectoral girdle development and myology in extant archosaurs^23^, the sterno-furcular complex in *H. escuilliei* supports the primary homology of the theropod furcula with the interclavicle^22,23^ and not with the clavicles^18^.

Several stem-avians (i.e., non-avian dinosauromorphs) bridge the morphological disparity between the sterno-interclavicular complex of extant birds and those of non-avian reptiles. The couple of incipiently-developed processes in the anterior end of the sauropod interclavicle is topographically equivalent to the epicleideal rami of the theropod furcula^22^, and suggests that a “Y”-shaped interclavicle is ancestral to saurischians and cannot be considered a derived condition of the bird-like theropods. The furcular hypocleideum is variably developed among the earliest-diverging theropod branches^15,16,19,20^, supporting its presence in the theropod last common ancestor and the direct derivation from the parasternal ramus of the interclavicle of most reptiles. The absence of the hypocleideum in some theropod lineages^18^ is thus interpreted as a derived condition acquired independently among some allosauroids, tyrannosauroids and dromaeosaurids. Although in most theropods the furcula is reconstructed as being separated from the sternum by a significant gap^14,18,21^, the actual extent of the sternal plates in most non-maniraptoran taxa is unknown^18,27,28,35^. In articulated maniraptoran specimens bearing the sternal complex at an advanced ossification grade, the posteroventral apex of the furcula closely approaches the anterior margin of the sternum, suggesting a connection between the two elements^18^. As in *Halszkaraptor*, oviraptorids and several ornithothoracine birds (i.e., some basal ornithuromorphs and most enantiornithines; e.g. *Concornis*^37^) bear a relatively elongated hypocleideum which directly contacts the ventral surface of the anterior end of the sternum^18,35–37^. This pattern suggests that when the elongate hypocleideum is present, the furcula bridges the scapulocoracoid with the sternum and is thus functionally equivalent to the interclavicle. Although in the majority of modern avians the furcula lacks an osseous articulation with the sternum^35–37^, in some taxa (e.g., *Fregata magnificens*; *Grus grus*; *Opisthocomus hoazin*; *Pelecanus crispus*, Fig. 5d,e) it is fused to the anteroventral margin of the sternal keel, along a suture which is topographically equivalent to the interclavicle-sternum contact present in *Halszkaraptor* and other diapsid reptiles. When not fused to the sternum, the hypocleideum of the majority of extant avians is linked by a ligament to the anteroventral surface of the sternum and shares with it the origin of the pectoralis muscle^23,34–36^. The latter originates from the interclavicle in both squamates and crocodiles^26,34^, providing additional support for the deep homology between that bone and the avian furcula: we infer that a ligamental connection between the hypocleidean end of the furcula and the anteromedian apex of the sternal complex (regardless of the latter being cartilagineous or variably ossified) was present in all theropods.

Although it is topographically homologous to the sterno-interclavicular contact of most reptiles, the bilobed sternal facet of the furcula in *H. escuilliei* is unreported among other non-avian theropods^18^. Its presence in *Halszkaraptor* might be related to the unusual adaptations inferred for this taxon. In particular, *Halszkaraptor* has been suggested to be a forelimb-assisted swimmer^32^, an adaptation which might require a more rigid pectoral apparatus compared to the sterno-furcular ligamental connection inferred for strictly ground-dwelling theropods^28^. Furthermore, *H. escuilliei* bears the longest neck relative to body size among Mesozoic paravians^32^: the stabilization of the trunk at the sterno-furcular junction might have provided a firm base for lateral movements of the markedly elongated neck, as also suggested for the development of the shoulder girdle in sauropods^22^.

**Figure 6:**
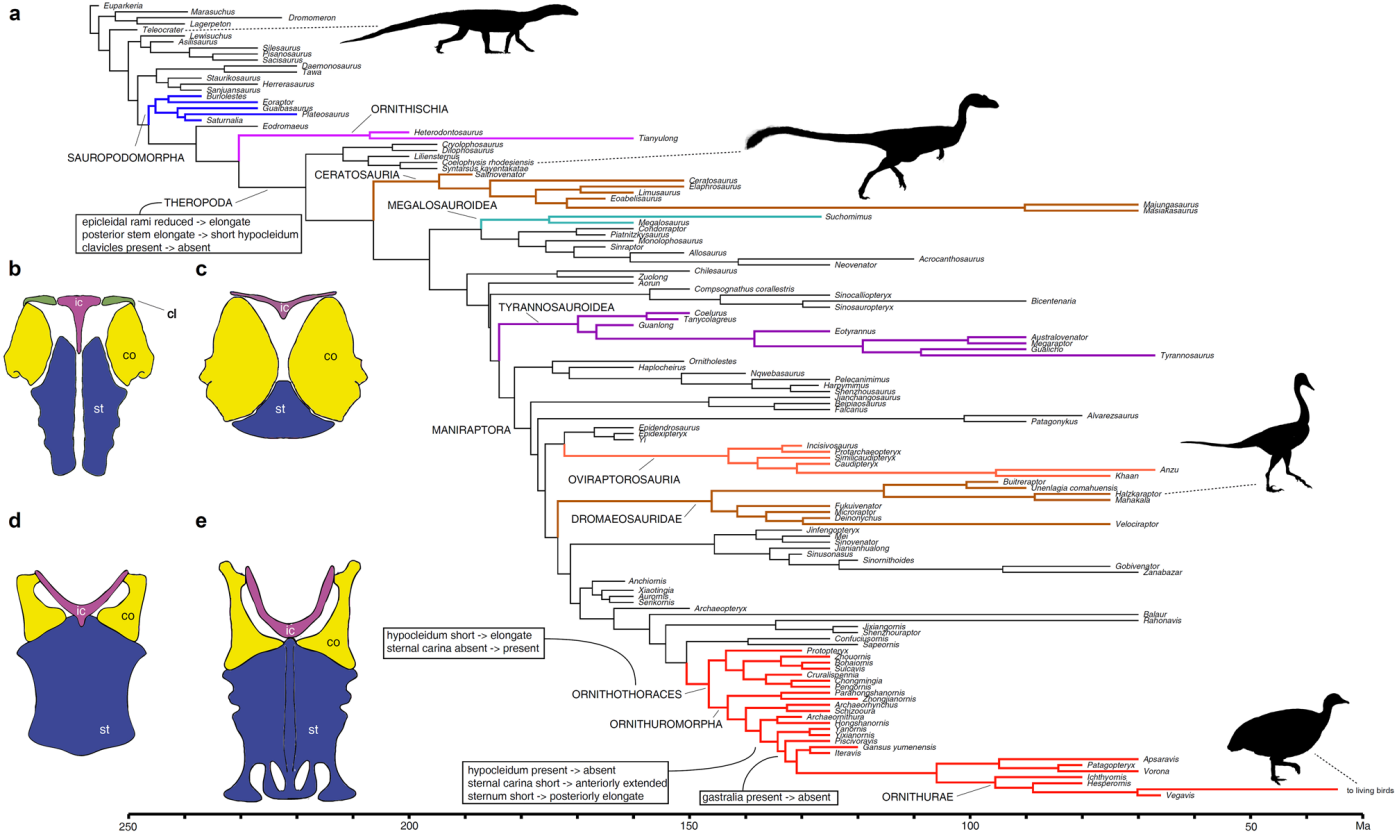
Evolution of the interclavicle and sternum along the avian stem lineage. (**a**) Time-calibrated phylogeny of Pan-Aves focusing on Theropoda^38^ with indicated the main morphological state transitions involving the interclavicle and the sternum^18,38^. Nodal optimisation under accelerated transformation. Colored branches indicate clades mentioned in the text. (**b–e**) Evolution of the pectoral apparatus along the avian-stem lineage (ventral view, scapula omitted for clarity). (**b**) Dinosaurian ancestral pattern; (**c**) early theropod pattern; (**d**) pennaraptoran pattern; (**e**) ornithuromorph pattern. *cl* clavicle, *co* coracoid, *ic* interclavicle, *st* sternum. Drawing by AC, with silhouettes modified from previous studies^1,32,41^.

The interpretation of the wishbone as the interclavicle^23^ removes the furcula from the series of theropod novelties^18,38^; instead, the pertinent novelty is the loss of the clavicles (Fig. 1b). Under this scenario (Fig. 6), the furcula originated directly from the reptilian interclavicle through elongation of the epicleideal extremities and the reduction of the posterior stem to a small hypocleideal tuberosity, resulting in the acquisition of a bilateral scapular contact and the loss of the direct osseous contact with the sternum. We suggest that the evolution of the bipedal predatory *bauplan* of theropods from the ancestral quadrupedal archosaurs was achieved through reinforcing the interconnection between the contralateral scapulocoracoids and by relaxing the bridge between the latter with the sternum^14–20,28,38^. The function of the forelimbs in theropod ancestors shifted from being component of the locomotor quadrupedal module to an independent module contralaterally integrated and specialized to grasping^4,18,28,38^. The loss of the clavicles led the interclavicle lateral rami to enlarge and contact the scapular acromia producing a transverse embracing between the contralateral scapulocoracoids more stable than the one produced by the paired clavicles (and retained in early sauropodomorphs^24^). In the strictly bipedal theropods, the interclavicle reduced the stem-like posterior ramus to the tuber-like hypocleideum and lost the direct osseous contact with the sternal plates, replaced by a ligamental connection. The later evolution of the forelimb locomotor module among maniraptoran theropods (e.g., for active flight^4,6–9,12,13^ or forelimb-assisted swimming^32^), drove the re-acquisition of a tighter connection between the scapulocoracoids and the sternal complex through the interclavicle.

## Methods

The *Varanus storri* specimen (BE-RBINS-VER-10900; Fig. 5a–c) was scanned using a RX EasyTom150 (RX Solutions, Chavanod, France; http://www.rxsolutions.fr), with an aluminum filter. Images were generated at a voltage of 110 kV and a current of 440 μA, with a set frame rate of 12.5 f/s and 10 average frames per image. This generated 1440 images and a voxel size of 50.9 μm. Reconstruction was performed using X-Act software from RX Solutions. Segmentation, visualization, and analysis were performed using Dragonfly software, Version 4.0 for Windows (Object Research Systems (ORS) Inc, Montreal, Canada, 2020; software available at http://www.theobjects.com/dragonfly).

Scans of the *Halszkaraptor escuillei* specimen (MPC-D102/108) were performed at the BM05 beamline of the ESRF using a FReLon 2K14 camera in association with a scintillating fiber for setting a pixel size of 53.58 µm. The beam was set with 0.4 mm of molybdenum and 12 bars of 5 mm of aluminium to reach an average energy of 110 keV. A propagation distance of 4 m was used. Scans were performed in half-acquisition mode (850 pixels) with 5000 projections and an exposure time of 0.2 s for each projection over 360°. Raw data were reconstructed using single distance phase retrieval as well as metallic oxide inclusions correction and texture enhancement algorithm. Segmentation and visualization were conducted using VGStudio 2.2 (Volume Graphics, Heidelberg, Germany).

The following avian skeletons were examined first-hand: *Fregata magnificens* BE-RBINS-VER-93143-A61, *Grus grus* BE-RBINS-VER-97.015-A1, *Pelecanus crispus* BE-RBINS-VER-83834.

The analysis of the morphological features describing the evolution of the interclavicle and sternum along the avian stem lineage was performed in TNT^39^ using a well-sampled phylogenetic data set (Supplementary Information) which focuses on the evolution of the avian body plan^38^. We enforced the time-calibrated topology^38^ as framework for character state transition optimization. In the topology^38^, we replaced the terminal units *Berberosaurus* and *Eustreptospondylus* with, respectively, *Saltriovenator* and *Suchomimus* in order to provide information on the furcular morphology in Ceratosauria and Megalosauroidea^15,16^*.* Additional scores relative to the pectoral apparatus were optimized for the ornithischian^22^ and sauropodomorph^24^ branches or based on personal observation on the *Iguanodon bernissartensis* material housed in the RBINS. The ambiguous state transitions were optimized using WinClada^40^ and minimizing convergence over reversal (“fast optimization”). Institutional abbreviations: MPC, Institute of Paleontology and Geology, Mongolian Academy of Sciences, Ulaanbaatar, Mongolia. RBINS, Royal Belgian Institute of Natural Sciences, Belgium.

## Supplementary Information

Supplementary Information 1.

Supplementary Information 2.

## Data Availability

The synchrotron data used in this study are available on the European Synchrotron Radiation Facility open access database at http://paleo.esrf.fr/index.php?/category/2102. The µCT data of the *Varanus storri* specimen used in this study are available at RBINS upon request to J. Brecko (jbrecko@naturalsciences.be). The data matrix and time-calibrated topology are included in the Supplementary Information.
