# Supplementary material for: Unusual pectoral apparatus in a predatory dinosaur resolves avian wishbone homology
Source: Sci Rep. 2021 Jul 19;11:14722. doi: 10.1038/s41598-021-94285-3 (PMC8289867; doi:10.1038/s41598-021-94285-3)
Supplement: Supplementary file 2 — Supplementary Information 2. [file 41598_2021_94285_MOESM2_ESM.doc]

**Supplementary Information of**

**Unusual pectoral apparatus in a predatory dinosaur resolves avian wishbone homology**

Andrea Cau*, Vincent Beyrand, Rinchen Barsbold, Khishigjav Tsogtbaatar, & Pascal

Godefroit

**Character statements describing the morphological features of the interclavicle, sternum and gastral basket (complete character list from Ref. 38)**

**Interclavicle/furcula**

Furcula, width: less (0); more (1) than 4/5 of proximal scapular width. (Char. # 1384).

Furcula, shape: “V”-shaped (0); “U”-shaped (1). (Char. # 1398).

Hypocleideum (interclavicle posterior ramus): absent (0); present (1). (Char. # 1385)

Hypocleideum, length: less (0); more (1) than 1/3 epicleideal rami length. (Char. # 1386).

Hypocleideum, cross section: rounded (0); keeled (1). (Char. # 1133).

Interclavicular angle: more than (0); less than (1) 70°. (Char. # 341).

Omal tips, shape: straight (0); curved dorsally (1). (Char. # 1294).

Omal tips, expansion in lateral view: absent (0); present (1). (Char. # 1295).

Lateral longitudinal groove: absent, epicleideum oval in cross section (0); present, epicleideum “V” or “L” shaped in cross section (1). (Char. # 653).

Furcular symphysis, cross section: rounded (0); anteroposteriorly compressed (1). (Char. # 1134).

**Sternum**

Paired plates, ventral surface: less than or subequal to (0); more than (1), that of the coracoids. (Char. # 1038).

Plates in articulated adult specimens: unossified (0); ossified (1). (Char. # 1037).

Sterna: paired (0); fused into a single element (1). (Char. # 335).

Costal processes, number: 3 or less (0); 4 or more (1). (Char. # 1376).

Carina: absent (0); present (1). (Char. # 1373).

Carina, anteroposterior extent: limited to the posterior half (0); extended to almost the whole length (1) of the sternum. (Char. # 1374).

Anteroposterior length: less than 3/2 (0); more than 3/2 (1) of the mediolateral width of the anterior half. (Char. # 701).

Anterior margin of the paired sterna, shape in ventral/dorsal view: concave to straight (0); distinctly convex (1). (Char. # 336).

Anterior margin in taxa with medially fused plates, rostral spine: absent (0); present (1). (Char. # 1739).

Articular facet for coracoid (conditions may be determined by the articular facet on coracoid in taxa without ossified sternum), position: anterolateral or more lateral than anterior (0); almost anterior (1). (Char. # 337).

Prominent anterolateral processes: absent (0); present (1). (Char. # 702).

Lateral margin, shape: convex to straight (0); broadly concave at mid-length (1). (Char. # 579).

Sternum, posterolateral processes: absent (0); present (1). (Char. # 338).

Sternum, posterolateral processes, elongation: do not reach (0); do reach (1) the posteriormost extent of the posteromedian (xiphoid) process of the sternum. (Char. # 1577).

Posterolateral process, distal end, shape: unexpanded (0); with distinct mediolateral expansion (1). (Char. # 654).

Posterolateral process, distal expansion (when present), shape: triangular/fan-shaped (0); forked/branched (1). (Char. # 1716).

Posterolateral process, inclination: parallel or nearly parallel to the long axis of the sternum (0); directed laterodistally so that the distal ends are located lateral to the anterior half of the sternum (1). (Char. # 1297).

Posterior margin in taxa bearing a distint posteromedian process, shape in ventral view: medially acuminate (0); flat to convex, mediolaterally expanded (1). (Char. # 1428).

Posteromedian process in taxa with posteriorly acuminate sternum, proximodistal length: no more than (0); more than (1) 3/2 of its proximal mediolateral width (0). (Char. # 340).

Posteromedian process in taxa with posteriorly acuminate sternum, distal end, shape: tapering (0); expanding mediolaterally (1). (Char. # 904).

Paired posteromedial processes, development: indistinct (0); distinctly elongate (1). The lateral margin of the posterior sternal fenestrae – when present (Char. # 903) – is considered formed by the posteromedial process. (Char. # 339).

Paired posteromedial processes, posteromedial contact with median process enclosing two fenestrae: absent (0); present (1). (Char. # 903).

**Gastralia**

Gastralia: ossified (0); absent or unossified (1). (Char. # 248).

Number of sets of fused medial elements: zero or one (0); greater than one (1). (Char. # 956).

Medial element, distal end, shape: tapered (0); club-shaped prominence (1). (Char. # 955).

**Additional score for the sauropodomorph branch (Ref. 22 and 24):**

1385.1, 1386.1

**Additional score for the ornithischian branch (pers. obs, in prep.):**

1385.1, 1386.1
